# Editorial: Recovering after terrorist attacks, large-scale accidents and other disasters: Psychosocial care responses across countries

**DOI:** 10.3389/fpsyg.2023.1168679

**Published:** 2023-03-22

**Authors:** Lise Eilin Stene, Arnaud Fernandez, Morgane Gindt, Ophélie Nachon, Florence Askenazy

**Affiliations:** ^1^Norwegian Centre for Violence and Traumatic Stress Studies, Oslo, Norway; ^2^Service Universitaire de psychiatrie de l'enfant et de l'adolescent, Hôpitaux pédiatriques de Nice, Centre Hospitalier Universitaire-Lenval, Nice, France; ^3^CoBTeK (Cognition-Behaviour-Technology) Lab, Université Cote d'Azur, Nice, France

**Keywords:** psychosocial care, terrorist attacks, mass casualty incidents, disasters, posttraumatic stress disorder, posttraumatic health problems

Planning psychosocial care is essential to efficiently respond to and recover from mass casualty incidents, such as terrorist attacks, accidents and natural disasters. Since such incidents are typically unpredictable and often require urgent responses under chaotic circumstances, it is challenging to organize an adapted psychosocial care response to identify and follow-up affected individuals who need psychosocial care interventions. A large number of people may be impacted, including those directly exposed; professional or volunteer first responders; people living or working nearby; family members or friends of the survivors and the bereaved. Thus, it may be difficult to identify and reach the target population(s), even if some clinical and environmental factors that may influence the risk of developing posttraumatic health problems have been described in scientific literature (Brewin et al., 2000; Trickey et al., 2012). In order to prevent or treat posttraumatic health problems in affected populations, it is essential to learn from good practices and the best available evidence. Unfortunately, existing research is scarce and international guidelines on post-disaster psychosocial care are largely based on expert consensus (Bisson et al., 2010; Te Brake and Dückers, 2013; EU Handbook on Victims of Terrorism, 2021). Furthermore, little is known about how different countries actually meet psychosocial care needs after man-made and natural disasters. This knowledge is essential in order to strengthen the public health preparedness to disasters internationally and transnationally.

Considering the scarcity of studies in this field, we proposed a Research Topic with the goal to generate knowledge and accumulate experiences on psychosocial care responses to terrorist attacks, large-scale accidents and other disasters across different settings, populations and countries. There is an urgent need for interventions that can be implemented in humanitarian settings in areas with limited healthcare resources and where armed conflicts and disasters are prevalent. In this context, the study of Ahmadi et al. provided promising findings on a low-intensity and community accessible intervention with modified written exposure therapy to reduce posttraumatic stress symptoms. This pilot randomized control trial was conducted in Afghanistan and included adolescent girls exposed to a terrorist attack. If future studies also find similar favorable results for this type of low-intensity therapy in different population groups, the intervention could be delivered by personnel with minimal training and potentially be made widely available for communities with limited healthcare facilities.

In a quite different context, namely in Nice, France, the study of Gindt et al. underscored that also in countries with universal health coverage and well-developed health systems, the mental health impact of a terrorist attack was severe and long-lasting among survivors—including children and adolescents. Gindt et al. found a high rate of posttraumatic stress disorder (PTSD) and comorbidities, such as sleep disorders and mood and anxiety disorders. Furthermore, many children and adolescents needed follow-up several years after the attack. Sometimes first-time consultations even occurred more than 3 years after the attack.

Moreover, the longitudinal study of Bosmans et al. focusing on the Utrecht tramway shooting in the Netherlands, further documented that the survivors of the terrorist attack commonly experienced long-term health problems and encountered difficulties in finding appropriate healthcare. Additionally, the study unveiled that the implementation of a post-attack public health monitor was accompanied by multiple challenges, highlighting that improved practices for registration of survivors and exchange of their contact information are needed. Accessing such relevant data in order to track evolving psychosocial needs was also reported as a challenge in another study from the Netherlands by van Herpen et al. evaluating the users' and the service providers' experiences with the Information and Referral Center (IRC). The IRC was a one-stop shop concept for disaster response services established after the MH17 airplane crash, affecting many Dutch citizens. Despite the challenges of tracking psychosocial needs, both the users and the providers of services were positive about the merits of the IRC. The users perceived the IRC as a reliable information source and appreciated the possibility for further referrals if needed, while the involved organizations reported that the IRC helped to structure and align their services.

Finally, in order to respond to the needs and rights of survivors of mass trauma, it is crucial to provide proper training and preparedness of professionals involved in the psychosocial care response. As addressed in the perspective paper by Askenazy et al., there is a recognized lack of inter-professional and international trainings in the context of terrorist attacks in Europe. This is important also in order to provide adequate help to those affected by terrorist attacks or similar mass trauma in other countries than their home country. The paper reported perspectives from an expert panel covering the pertinence of remote trainings for professionals involved in the acute response of a terrorist attack. They concluded that, even though remote trainings cannot replace in-person trainings, they may be beneficial to share knowledge on the role and organization of different types of professionals and potentially improve the response coordination and share good practices across different disciplines and countries.

The Research Topic included articles addressing both civilians in need of acute and long-term psychosocial care and providers of psychosocial care after terrorist attacks and other types of disasters. The studies covered different geographical settings and yielded new and important insight that may help strengthen future disaster preparedness and psychosocial care responses. Yet, an urgent need remains for methodologically sound studies conducted in a way that will allow for cross-country comparisons and sharing of good practices. Special attention must be paid to at-risk populations, children and adolescents, the elderly, and people with cognitive or physical disabilities. This is essential in order to strengthen our capacities to address the health needs of individuals and communities impacted by mass casualty events.

## Author contributions

LES wrote the original draft of the editorial. AF, MG, ON, and FA contributed to the drafting and discussion of the editorial. All authors contributed to the article and approved the submitted version.
